# The deleterious effect of bisphenol S on early embryo development of mice

**DOI:** 10.1007/s42000-025-00638-2

**Published:** 2025-03-04

**Authors:** Christina Mantzouki, Despoina Mavrogianni, Maria Papagianni, George Konomos, George Creatsas, Peter Drakakis, George Mastorakos

**Affiliations:** 1 Unit of Endocrinology, Diabetes Mellitus and Metabolism, Aretaieion Hospital, Athens Medical School, National and Kapodistrian University of Athens, 76 Vas. Sofias Str., Athens, 10674 Greece; 2 1st Department of Obstetrics and Gynecology, “Alexandra” General Hospital, School of Medicine, National and Kapodistrian University of Athens, Athens, Greece; 3 Department of Nutrition and Dietetics, School of Physical Education, Sport Science and Dietetics, University of Thessaly, 42100 Trikala, Greece; 4 LE MONDE College, Athens, Greece; 5 Medical School, National and Kapodistrian University of Athens, Athens, Greece; 6 3rd Department of Obstetrics and Gynecology, “Attikon” General Hospital, School of Medicine, National and Kapodistrian University of Athens, Athens, Greece

**Keywords:** Bisphenol S, Endocrine disruptor, Embryos, Female reproduction, Embryo development

## Abstract

**Purpose:**

Increasing levels of infertility in Western countries has drawn ever more scientific attention to the role in this trend of endocrine disruptors, such as bisphenol A, a substance now banned in some cases and some countries. Because this substance has been replaced by the structurally similar bisphenol S (BPS), this study focused on the effects of the latter on early mice embryo development.

**Methods:**

Cultures of CD1 mice embryos with varying concentrations of BPS were compared with control blank cultures in order to examine the survival rate of embryos according to BPS concentration and culture day.

**Results:**

The administration of BPS at any dose (1, 10, and 100 pg/ml) in cultures of mice embryos led to a significant decrease in their survival rate. The negative effect of BPS was seen to start early (day 1 of experiment), even with the lowest employed dose (1 pg/ml).

**Conclusions:**

This is, to the best of our knowledge, the first study to investigate the impact of BPS on the survival rate of mice embryos. In this study, potential adverse effects of BPS on early CD1 mice embryo development with regard to survival rate have been identified. Dose of BPS, timing of BPS administration, and time duration of exposure play a critical role in the decrease of mice embryo survival rate as compared to control cultures. These findings raise concerns regarding the safety of BPS and highlight the need for further research into the effect of this substance on human embryos.

## Introduction

Increasing infertility rates in Western countries have led researchers to scrutinize the role of environmental factors, such as endocrine disruptors, in this trend [1]. One of the commonest endocrine disruptors, for the past 40 years, is bisphenol A (BPA) which has been related to numerous serious implications for public health as well as for the environment [2]. For this reason, its production and use have been partially banned in some countries by global regulatory agencies over the last few years. It has, however, subsequently been replaced in industry by structurally similar but less known alternative chemicals such as bisphenol S (BPS) [3–5].

Both BPA and BPS enter the human body via ingestion, inhalation, or dermal contact and are rapidly metabolized and gradually excreted [6, 7]. BPS is being used increasingly in a wide array of consumer and industrial products [8, 9], its use in thermal paper having increased by 153% between 2018 and 2019 [10]. Recent human biomonitoring studies revealed the presence of BPS in 89.4% of urine samples from the general U.S. population, and in 67.8% of urine samples from a large population (*n* = 1396) of pregnant women in the Netherlands [11, 12]. Recent data have shown that BPS exposure in the general population ranges from 0.01 to 10 ng/mL in several types of body fluids, including blood, urine, breast milk, and follicular fluid [13–15]. In humans, BPS is metabolized in the liver into BPS-glucuronide and BPS-sulfate and finally excreted in urine and feces, while it is detected in urine at similar concentrations as BPA (0.02-21 ng/mL or 0.09-91 nM) [9, 16].

Similarly to BPA, BPS has also been shown to possess estrogen- or antiandrogenic-like properties and can bind to estrogen or androgenic receptors [17]. Thus, a few studies have already demonstrated that BPS exerts endocrine disrupting effects impairing reproduction in several species [18]. In Zebrafish, 75 days of BPS exposure is reported to increase plasma estradiol levels and decrease egg production and sperm count [19]. In female mammals, BPS disrupts the estrous cycle, folliculogenesis, and early developmental oocyte competence [20]. Furthermore, recent toxicological investigations in zebrafish, rodents, and sheep have revealed that exposure to BPS during development can disrupt reproductive, metabolic, and developmental endpoints through the endocrine pathways, indicating the vulnerability of the gestational period to BPS exposure [19, 21–26]. In fish and rodent species, BPS affects germinal cells and endocrine function similarly to BPA [27]. Regarding BPA, several studies have described a negative association of BPA concentrations with embryo quality, cleavage, and blastocyst rates as well as embryo cell number [28–33], while BPA was reported to disrupt blastocyst formation in vitro [34]. Recently, BPS was found in four maternal (0.03–0.07 ng/mL) and seven cord serum samples (0.03–0.12 ng/mL) from a population of 61 mother-newborn pairs in China, providing the first evidence that BPS crosses the human placenta [35]. In addition, BPS was detected in maternal serum (0.01 ng/mL) and cord serum (0.03 ng/mL) in 106 maternal-fetal pairs in an e-waste dismantling site in Southern China [36].

Over the last few years, there have been considerable efforts by the scientific community to evaluate the safety of BPS since most people are regularly exposed to this molecule through their diet, though also via numerous products and in dust. However, data regarding the toxic effects of BPS on embryo development are still lacking. To investigate the role of BPS in an in vitro model of mouse embryo development, the effect of the administration of different concentrations of BPS in cultures of mice embryos was evaluated and the survival rate of embryos according to BPS concentration and culture day was examined.

## Materials and methods

### Animals

Four-week-old CD1 female and 7- to 9-week-old male mice were housed in standard polysulfonate cages and maintained in an animal facility in the following conditions: 12 h light/12 h darkness photoperiod; 22 ± 2 °C and 40–70% relative humidity. A phytoestrogen-free diet 1814P (Altromin, AnimaLab, Poznan, Poland) and ultrapure water (in glass bottles) were provided ad libitum. The animals were allowed to acclimate for at least 1 week prior to initiation of treatment. All animal procedures complied with PD 56/2013 and the European Directive 2010/63/EU, welfare and ethical use of laboratory animals based on the 3R’s: Replacement, Reduction and Refinement and the guidelines of PREPARE (Planning Research and Experimental Procedures on Animals: Recommendations for Excellence), and ARRIVE (Animal Research: Reporting in Vivo Experiments) [37–40]. The maintenance of the laboratory mice and the in vivo studies were performed in specific pathogen free conditions at the approved establishments of the Department of Animal Models for Biomedical Research, Hellenic Pasteur Institute, Athens, Greece, under the registered codes EL25BIO011, EL25BIO012, and EL25BIO013. The animal study was reviewed and approved by the Committee for Evaluation of Experimental Procedures, Department of Experimental Animal Models, Hellenic Pasteur Institute.

### Materials

Pregnant mare serum gonadotropin (PMSG) was purchased from BioVendor LLC (Karásek, Brno, Czech Republic). IVF medium was purchased from Origio Medicult Media (Ballerup, Denmark). Bisphenol S and human chorionic gonadotropin (hCG) lyophilized powder were purchased from Sigma-Aldrich Chemie GMbh (Shanghai, China), while BPS concentrations were prepared with serial dilutions from a stock solution of 1000 mg/L BPS (powder) in sterilized water. All other solutions (i.e., M2, M16, and Ham BSA) and paraffin oil were purchased from Merck KGaA (Darmstadt, Germany). Mice embryos were cultured in Petri dishes 60*15 mm (BD Falcon; Franklin Lakes, NJ).

### Protocol

#### Preparation of fertilized oocytes

Ovulation was induced in female mice aged 3 to 4 weeks old by injecting intraperitoneally 5 IU PMSG. Forty-eight hours later, hCG was injected to induce oocyte maturation, after which the female mice were mated with 10-week-old male mice. Mass production of mice embryos thereby took place. The breeding procedure was confirmed the next morning by the presence of a vaginal plug in the females. As ovulation occurs 11–14 h after hCG administration, 20 h following hCG administration, the fertilized mice were euthanized by cervical dislocation and their fallopian tubes were collected under sterile conditions. The fallopian tubes were then placed in a Petri dish with 1.5 mL M2 culture medium which contained 0.3 mg/mL hyaluronidase. Using a suitable stereoscope and special forceps, the swollen point of the fallopian tube (ampulla) was excised and the fertilized eggs were released into the culture medium. Following this, they were washed twice with the same culture medium containing hyaluronidase so that the cumulus cells could be mechanically removed.

#### Cultures of fertilized eggs with BPS

Subsequently, the stripped (washed) fertilized eggs were placed in culture dishes containing culture medium with 10% Ham and 10% BSA. The zygotes remained only briefly in the medium, after which the stripped embryos were transferred to a new dish containing M2 medium in order to be rinsed and to remove the last traces of hyaluronidase. They were then transferred onto the small control surface of a dish with the administration of four drops of M16 to ensure close proximity of zygotes and, subsequently, were covered with paraffin oil to avoid evaporation. The dish was placed in an incubator with the following culture conditions: 37^o^C temperature, 5% CO2, and 95% relative humidity. All solutions employed, which had been tested for use in mice embryos, were brought to room temperature. The embryos were cultured in Petri dishes using IVF universal medium. To evaluate BPS effects in vitro, it was important to consider the standardized lowest dose that would cause an adverse effect, also known as the *lowest observed adverse effect level* (LOAEL). To the best of our knowledge, an in vitro LOAEL dose of BPS has not yet been calculated. It should be noted that the LOAEL for BPA is reported to be 50 mg/kg/day for in vivo doses, extrapolated to an in vitro 0.05 mg/ml dose in mice [41]. The administrated BPS doses (1, 10, and 100 pg/ml) in this study are lower than the LOAEL for BPA. The embryos (*n* = 332) were randomly separated into four experimental groups exposed to four BPS concentrations (0, 1, 10, and 100 pg/ml) [hereafter designated as BPS0 (*n* = 75), BPS1 (*n* = 79), BPS10 (*n* = 105), and BPS100 (*n* = 73)], cultured until the blastocyst stage (day 4) under microscope observation. BPS concentrations were administered separately to cultures on day 0 and their effect on mouse embryo development was recorded. Specifically, embryo development was assessed daily under the microscope for 5 days (day 0 to day 4 of the experiment) through all stages of embryo development (i.e., zygote; 2-cell embryo; 4-cell embryo; morula; and blastocyst). Maturation was considered to be completed when blastocyst occurred. The experiments were repeated three times. The experimental protocol was executed in a university laboratory designed for in vitro fertilization.

#### Quantitative and qualitative evaluation of embryo by microscopy

During the culture period, the mice embryos were observed by microscope on a daily basis for a period of 5 days (embryo development analysis), from the zygote to the blastocyst stage. On day 1, oocytes with two pronuclei and two polar bodies were considered fertilized. Quantitative daily evaluation included calculation of the total number of surviving embryos and of the total number of blastocysts. Survival rate is the ratio of healthy embryos on each day of embryo development to the number of embryos on day 0. Qualitative daily evaluation was performed based on cytoplasmic determinants and nuclear-cytoplasmic ratio during the developmental stages. Embryos were considered of good quality if four blastomeres on day 2 or if six to eight blastomeres on day 3 were observed; this also applied if they had symmetric blastomeres, low percentage of fragmentation with clear cytoplasm and no intracytoplasmic abnormalities (vacuoles and inclusion bodies), no extracytoplasmic abnormalities, and if their cleavage corresponded to the development day. Embryos were considered of low and poor quality if cells were asymmetrical, cytoplasmic irregularities were observed, and the cleavage did not correspond to the embryonic development day. The rule is that on day 4, blastocysts with an inner cell mass and a blastocelic cavity should be seen. The quantitative and qualitative evaluation of embryos was performed by two independent scientists (CM and DM). There was no significant discrepancy between their evaluations.

### Statistics

The two-way analysis of variance (ANOVA) test was employed to determine the effect of concentration and day variables deviation (number of surviving embryos). Fisher’s exact test was used for comparisons of survival rates in different BPS concentrations and days of cultures. In order to examine the deviation from normality assumption, an Anderson-Darling test of normality was applied. Moreover, to enhance the conclusions derived from the two-way ANOVA, a non-parametric test (Friedman’s test) was employed. Statistical analyses were performed using SPSS software version 20 and Minitab version 16. Statistical significance level was set at 5%.

## Results

### Survival rates of embryos per culture day according to BPS concentration (Table 1)

On day 0, the number of embryos introduced to embryo cultures per BPS concentration was 75, 79, 105, and 73 in the BPS0, BPS1, BPS10, and BPS100 groups, respectively. In Table 1, absolute numbers of embryos as well as survival rates for each BPS concentration on each culture day are reported together with statistically significant differences between survival rates. The effects of each BPS concentration on each different day are described separately. At all concentrations employed, the survival rate started declining significantly past day 1, while at concentration BPS 100, no embryo survived past day 3. On day 1, survival rates of embryos decreased significantly compared to day 0 for BPS1, BPS10, and BPS100 (*p*-value < 0.001 for all comparisons, respectively) but did not for BPS0; on day 2, survival rates of embryos decreased significantly compared to day 0 for BPS1, BPS10, and BPS100 (*p*-value < 0.001 for all comparisons, respectively) but did not for BPS0; on day 2, survival rates of embryos decreased significantly compared to day 1 for BPS1, BPS10, and BPS100 (*p*-value < 0.001, *p*-value = 0.006, *p*-value = 0.012, respectively) but did not for BPS0; on day 3 survival rates of embryos decreased significantly compared to day 0 and day 1 for BPS0, BPS1, BPS10, and BPS100 (*p*-value < 0.001 for all comparisons, respectively); no significant differences in survival rates of embryos were observed between day 3 and day 2 for BPS0, BPS1, BPS10 and BPS100; on day 4 survival rates of embryos decreased significantly compared to day 0 and day 1 for BPS0, BPS1, BPS10, and BPS100 (*p*-value < 0.001 for all comparisons, respectively); on day 4 survival rates of embryos decreased significantly compared to day 2 for BPS0, BPS1, and BPS10 (*p*-value < 0.001, *p*-value < 0.007, *p*-value < 0.033, respectively) but did not for BPS100; and on day 4 survival rates of embryos decreased significantly compared to day 3 for BPS0 and BPS1 (*p*-value = 0.031, *p*-value < 0.001, respectively) but did not for BPS10 nor for BPS100.

**Table 1:** Number of mice embryos and their survival rate (in percentages) at all concentrations employed (from day 0 to day 4). The daily mice embryo survival rate refers to day 0 absolute number of embryos. Statistical significance was calculated using Fisher’s exact test and was set at *p*-value < 0.05. The asterisk (*), the dagger (†), the dollar sign ($), and the pound sign (#) indicate a statistically significant difference from day 0, day 1, day 2, and day 3, respectively. Point wise 95% confidence intervals are displayed in brackets

BPS concentration			Day of culture		
	Day 0	Day 1	Day 2	Day 3	Day 4
BPS0 (0 pg/ml)	75 (100%)	75 (100%)	71 (94.7%) [0.896; 0.998]	64 (85.3%) ^*, †^ [0.772; 0.934]	52 (69.3%) ^*, †, $, #^ [0.588; 0.798]
BPS1 (1 pg/ml)	79 (100%)	51 (64.6%) ^*^ [0.541; 0.751]	25 (31.6%) ^*, †^ [0.213; 0.419]	14 (17.7%) ^*, †^ [0.093; 0.261]	10 (12.7%) ^*, †, $, #^ [0.055; 0.199]
BPS10 (10 pg/ml)	105 (100%)	41 (39.0%) ^*^ [0.297; 0.483]	22 (21%) ^*, †^ [0.132; 0.288]	16 (15.2%) ^*, †^ [0.083; 0.221]	10 (9.5%) ^*, †, $^ [0.039; 0.151]
BPS100 (100 pg/ml)	73 (100%)	15 (20.5%) ^*^ [0.112; 0.298]	4 (5.5%) ^*, †^ [0.0026; 0.1070]	0 (0.0%) ^*, †^	0 (0.0%) ^*, †^

### Survival rates of embryos per BPS concentrations according to each culture days (Fig. 1)

**Fig. 1:**
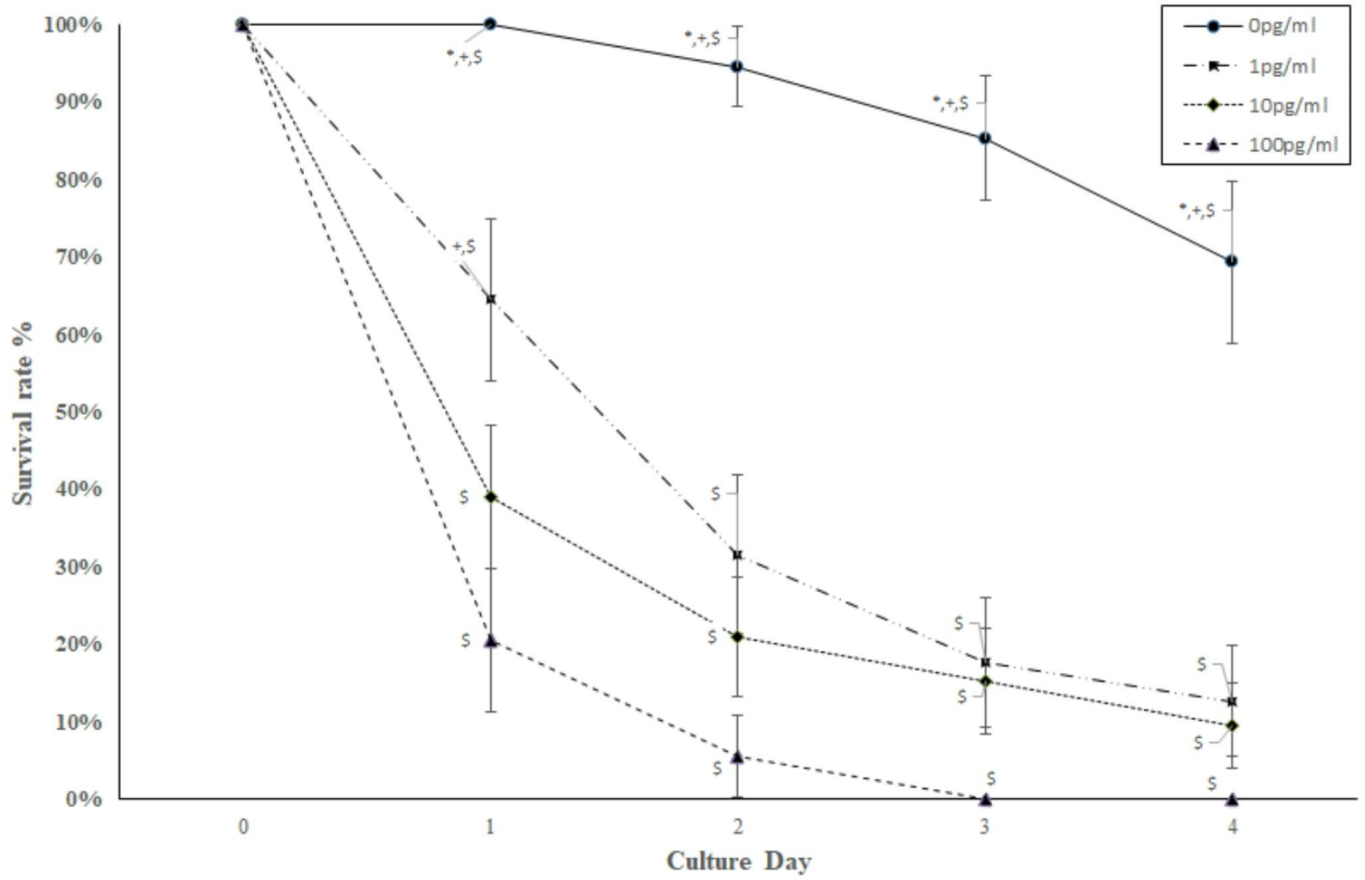
Statistically significant differences among different concentrations according to each culture day. Statistical significance was calculated using Fisher’s exact test and was set at *p*-value < 0.05. Point wise 95% confidence intervals are shown. The asterisk (*) indicates a statistically significant difference from concentration 1 pg/ml (on the same culture day); the cross (+) indicates a statistically significant difference from concentration 10 pg/ml (on the same culture day); the dollar sign ($) indicates a statistically significant difference from concentration 100 pg/ml (on the same culture day)

In Fig. 1, the effects of each BPS concentration on each culture day are compared with each other. On all days, survival rates for BPS0 were statistically greater than survival rates for BPS1, BPS10, and BPS100 (*p*-value < 0.001 for all comparisons). On day 1, survival rate for BPS1 was statistically greater than survival rates for BPS10 and BPS100, whereas on days 2, 3, and 4, survival rates for BPS1 were statistically greater than survival rates for BPS100 (*p*-value < 0.001 for all comparisons, respectively) but not for BPS10. On all days, survival rates for BPS10 were statistically greater than survival rates for BPS100 (*p*-value < 0.001 for all comparisons, respectively).

### Mean survival rate of embryos according to concentration of BPS and day of culture designated as main effects

**Fig. 2:**
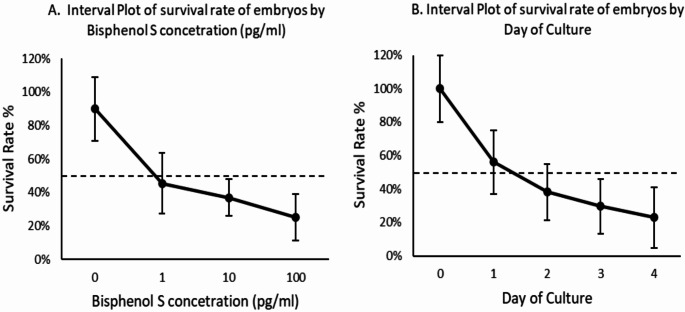
Main effects plot displaying the mean number of embryos by two-way ANOVA. Main effects: (**A**) Concentration of BPS. (**B**) Number of culture day. Dashed line indicates mean number of embryos. Point wise 95% confidence intervals are shown

A two-way ANOVA analysis revealed that the concentration of BPS (*p* = 0.002) (Fig. 2, A) and the number of culture day (*p* < 0.001) (Fig. 2, B) (both designated as main effects) combined, demonstrated a statistically significant effect on the survival rate of embryos. In detail, the two-way ANOVA analytical method was performed in order to examine the combined effects of the two factors acting simultaneously (1. BPS concentration; 2. day of culture) on the survival rate of embryos (value). By analyzing both factors together, the risk of type I error (false positive) is reduced. The latter might occur if multiple separate tests are run. Thus, a more accurate understanding of the main effects of each factor can be achieved as the analysis accounts for the variability due to both factors. The deviation from normality assumption was examined using the Anderson-Darling test of normality, which indicated that the residuals of the model were normally distributed (*p*-value = 0.099). The adjusted R-squared value for the two-way ANOVA model was 0.821, meaning that approximately 82.1% of the variability in the response variable (survival rate of embryos) can be explained by the two factors in the model. Moreover, to enhance our conclusions drawn from the two-way ANOVA, a non-parametric analysis of variance test (Friedman’s test) was employed, which does not assume any data derived from a normal distribution, and it led to the same statistically significant conclusions (concentration of BPS *p*-value = 0.022, day of culture *p*-value = 0.004). Hence, the non-parametric analysis of variance (Friedman’s test) confirmed the conclusions of the two-way ANOVA.

## Discussion

We found that the administration of different concentrations of BPS in cultures of mice embryos resulted in significant decrease of their survival rate. To the best of our knowledge, there is to date no study in the medical literature investigating the survival rate of mice embryos following administration of BPS in mice embryo cultures. In this study, the greatest BPS dose used in the mice embryo cultures was 100pg/ml, this being the one with the strongest negative effect of BPS on the survival rate of cultured embryos. In the mice embryo cultures of all BPS concentrations employed, an abrupt decrease in mice embryo survival rate was observed from day 0 to day 1, whereas no significant decrease was observed between day 2 and day 3, indicating that the deleterious impact of BPS on the cultured mice embryos takes effect early. It is noteworthy that in the BPS100 group, no mouse embryo survived after day 3, whereas in groups BPS1 and BPS10, a small percentage of mice embryos were still alive on day 4 (survival rate: 12.7% and 9.5%, respectively). In the BPS0 group (control group), a gradual decrease in the survival rate of mice embryos was observed from day 0 to day 4. This decrease is in line with the usually observed fate of cultured mice embryos in non-interventional conditions. In the present study, the time duration of exposure to BPS appears to play an important role in early embryo development.

In a recent study, Saleh et al. reported that administration of BPS (0.05 mg/mL) to cultures of bovine oocytes and zygotes led to increased DNA fragmentation in 2–4 cell, 8–16 cell, and blastocyst embryos (*p* < 0.05): they suggested that BPS can exert disruptive effects (increased embryonic arrest and apoptosis) during early embryo development [42]. Long-term exposure of female mice, via their drinking water, for 4 weeks to very low doses of BPS (0.001-100 nanogram/gram bodyweight/day) reduced embryo cleavage rate and blastocyst rate [43]. Exposure of mice embryos for 21 days to different doses of BPS (1.000–100.000 µg/bw/day intraperitoneally) caused developmental arrest in preimplantation embryos [44]. In a 2022 study, the BPS in vivo effects on folliculogenesis and embryo production after chronic low BPA exposure through diet and the influence of metabolic status were investigated in adult ewes [45]. Sixty primiparous 2.5 year-old ewes, well-fed or on a restricted diet, were exposed to BPS (0, 4–50 µg/kg/day) for at least 3 months. No effect of BPS was observed on follicle population, plasma AMH levels, and embryo production numbers and rates. However, a significant diet x BPS dose interaction was reported for cleaved embryos, > 4-cell embryos, blastocyst, and early blastocyst numbers. The significant diet x BPS dose interactions observed in embryo production suggested that the effect of BPS is modulated by metabolic status. Exposure of developing chicken (Gallus gallus domesticus) embryos, in ovo from embryonic days 5 to 12, to increasing concentrations of BPA, BPS, BPAF, and TMBPF (0.003-30 µM) was followed by severe dose-dependent growth and embryonic defects, even at low, physiologically relevant concentrations [46]. Of interest, BPAF and TMBPF are BPA alternatives of remarkably similar chemical structure to that of BPA. To our knowledge, no significant data have been published regarding the role of BPS in human embryo quality and survival rate [47].

BPA was reported to disrupt human and bovine blastocyst formation in vitro [34]. Acute exposure of bovine embryo cultures to an environmentally relevant BPA concentration (1 and 10 ng/mL) negatively affected early embryo development and metabolism when BPA was applied at the greatest concentration (10 ng/mL) [48]. Importantly, embryos exposed to an increased BPA dose (10 ng/mL) demonstrated an altered metabolic profile, with significantly increased glucose consumption, suggesting potential early perturbation of offspring metabolism. In another study, in vitro exposure of maturing bovine oocytes to BPA led to decreased embryo quality and developmental potential in a dose-dependent manner, while it affected gene expression of developmentally important transcripts [31]. Significantly, studies in various animal models and cell systems have reported that some analogs, such as BPAF, are even more toxic, potent, and estrogenic than the parent compound BPA [49–54]. It must be highlighted that, although these analogs received much less testing and oversight, they are already being mass-produced and used across industries in the manufacture of a wide range of products, from plastics to food-contact coatings.

Regarding humans, in a recent cross-sectional study including 93 women of reproductive age with polycystic ovary syndrome (PCOS) and women with tubal factor infertility (TFI) (PCOS: 45; TFI: 48) following IVF, we found that in the TFI women, follicular fluid BPA concentrations correlated negatively with the number of retrieved oocytes [55]. Meanwhile, only a few in vivo studies have examined the effects of BPA on human embryo development. In a prospective preconception cohort study of 174 women undergoing IVF, urinary BPA concentrations were associated negatively with the rate of normally fertilized oocytes [56]. Data from epidemiological studies demonstrated that combined exposure to BPA from dietary and non-dietary sources during pregnancy may contribute to abnormal fetal growth, especially when exposure occurs during the first half of pregnancy [57].

An additional finding of the current study was the progressively decreased survival rate of the cultured mice embryos on each culture day at all BPS concentrations used compared to that of the control mice embryos on the corresponding day, indicating that the administration of BPS to the cultured mice embryos at any dose employed exerts a significant negative effect on the survival rate of embryos. Notably, no significant decrease in the survival rate of the cultured mice embryos was observed on days 2, 3, and 4 between the cultures with the two lower concentrations (1 pg/ml, 10 pg/ml), indicating that the negative effect of BPS is similar with the use of either of them and it started early with the lowest employed dose in this study. However, the administration of BPS at the greatest dose employed (100 pg/ml) resulted in lower survival rates than those following the administration of the lowest dose employed (1 pg/ml) on all days. Furthermore, the administration of BPS at the greatest dose employed (100 pg/ml) resulted in lower survival rates than those following the administration of the intermediate dose employed (10 pg/ml) on all days. Thus, in the current study, the strongest negative effect of BPS on the survival rate of cultured embryos was observed with the administration of the 100 pg/ml dose. The combined effect of the concentration of BPS with that of the culture day exerted a significant effect on the survival rate of mice embryos (Fig. 2). While the effects of BPS on human reproductive health embryo quality are as yet largely unknown, the present data suggest that BPS has the potential to induce changes during embryo development in female mice. It thus appears evident that BPS is not a safer alternative to BPA.

To the best of our knowledge, an in vitro LOAEL dose of BPS has not as yet been calculated. The LOAEL for BPA was reported to be 50 mg/kg/day for in vivo doses and has been extrapolated to an in vitro 0.05 mg/ml dose in mice [41]. Administrated BPS doses in the present study are lower than the LOAEL for BPA. The BPS concentrations employed in this paper appear to be lower than those found in the general population.

Recent in vitro and in vivo data have demonstrated that BPS has equal or even greater toxicological effects than those of BPA, with an endocrine-disrupting effect similar, at least in part, to that of the latter [18, 58–62]. A recent assessment of the levels of BPS has shown that this compound is ubiquitously present in the environment and biota, this pointing to its prospective risk as an emerging contaminant [17]. For example, BPS has been detected at high concentrations in human serum (30-1500 ng/L), while it has additionally been found in water samples (1-169,000 ng/L) [63–64, 35, 17]. Interestingly, BPS concentrations in water samples from the Pearl River in China (135 ng/L) and the Adyar River in India (6840 ng/L) were considerably greater than those of BPA (73 and 359 ng/L for the two rivers, respectively) [65]. In sediments collected from the USA, Japan, and Korea, BPS has been detected in concentration ranges of 0.002–1970 ng/g dry weight, while in municipal sewage sludge collected in 20 provinces in China, the detected concentrations were in the range of 0.17–110 ng/g dry weight [66]. In addition, BPS has been reported in food products in concentration ranges of 0.005–0.609 ng/g fresh weight in the USA and up to 36.1 ± 4.4 ng/g fresh weight in canned foods from Spain, while it has been detected in indoor dust in parts of Asia (e.g., China, Japan, and Korea) within a range of 0.026–111 µg/g [67–69].

While the present study demonstrates that BPS affects mouse embryo development, one limitation is that a detailed analysis of biochemical analytes revealing the physiopathology of the phenomenon is lacking. The mechanism via which BPS affects embryo development is largely unknown, with neither the metabolic nor the biological fate of BPS having to date been fully examined. An in vitro study indicated that glucuronidation was a major metabolic pathway for BPS action [70]. Meanwhile, as a potential EDC, BPS has been examined in the National Toxicology Program Tox21 High Throughput Screening Program and was classified as an estrogen agonist with a weak affinity for the estrogen receptor (ER) [71, 72]. The endocrine activity of BPS was examined by comparing the estrogenic binding potency of BPS against ethinyl-estradiol as positive control, on a plethora of ERs. The average estrogenic potency of BPS to ER receptors was found to be similar to that of BPA [73], the authors of the latter study suggesting that the described estrogen activity of BPS impairs embryo development. A recent study demonstrated that exposure to high levels of estradiol (≥ 10^− 7^ M) in mice embryo cultures has a deleterious effect on early post-implantation embryonic development [74]. Furthermore, in the past, both BPA and BPS were shown to alter steroidogenesis by increasing estradiol secretion and reducing progesterone secretion, while BPS was more detrimental to progesterone secretion than BPA because it induced its reduction at a concentration ten-fold lower than that of BPA [75]. Moreover, it is possible that the embryo maturation stage is more sensitive to the impact of BPS at early stages of embryo development. Significantly, early developmental stages, particularly embryonic development, are a critical window for growth and genome programming [76]. In this line of research, another recent study reported that maternal BPS exposure affects the reproductive capacity of F1 and F2 offspring in mice [77]. The authors suggested that the timing of exposure to BPS is important for the expression of the BPS effect, which parallels the conclusions in the present study.

In summary, this study has determined that BPS appears to exert in vitro deleterious effects on early mouse embryo development. Thus, the administration of BPS in cultures of mice embryos at any dose employed exerts a significant negative effect on embryo survival. Even the lowest concentration of BPS employed (1 pg/ml) had a detrimental effect on embryo survival rate, while the strongest negative effect of BPS on the survival rate of cultured embryos in the current study was observed with the administration of the greatest dose used (100 pg/ml). Of note, the negative effect of BPS starts early (day 1 of experiment) even with the lowest employed dose (1 pg/ml).

In conclusion, BPS appears to negatively affect early mouse embryo development, with the BPS dose, the timing of its administration, and the time duration of exposure to it each seeming to play a critical role in the process. Further studies are required to investigate the effect of BPS on human embryos in order to reveal a possible deleterious impact of this endocrine disruptor on human fertility.

## Declarations

### Attestation statements

Data regarding any of the subjects in the study have not been previously published.

## References

[1] Amar S, Binet A, Téteau O, Desmarchais A, Papillier P, Lacroix MZ et al (2020) Bisphenol S impaired human granulosa cell steroidogenesis in vitro. Int J Mol Sci 21(5):1821 32155818 10.3390/ijms21051821 PMC7084356

[2] Almeida S, Raposo A, Almeida-González M, Carrascosa (2018) Bisphenol A: food exposure and impact on human health. Compr Rev Food Sci Food Saf 17(6):1503–1517 33350146 10.1111/1541-4337.12388

[3] ECHA (2017) Seven New Substances Added to the Candidate List entry for bisphenolA updated [WWW Document]. URL. https://echa.europa.eu/fr/-/seven-new-substances-added-to-the-candidate-list-entry-for-bisphenol-a-updated-to-reflect-its-endocrine-disrupting-properties-for-the-environment

[4] French government (2012) Le bisphenol A interdit dans les contenants alimentaires - Ministere de l’Environnement, de l’Energie et de la Mer [WWW Document].URL. http://www.developpement-durable.gouv.fr/spip.php? page = article&id_article = 30371

[5] USEPA (2014) Bisphenol A alternatives in thermal [WWW document]. URL. https://www.epa.gov/sites/production/files/2014-05/documents/bpa_final.pdf

[6] Wang L, Zhang Y, Liu Y, Gong X, Zhang T, Sun H (2019) Widespread occurrence of bisphenol A in daily clothes and its high exposure risk in humans. Environ Sci Technol 18(12):7095–7102 10.1021/acs.est.9b02090 31124657

[7] Oh J, Choi JW, Ahn YA, Kim S (2018) Pharmacokinetics of bisphenol S in humans after single oral administration. Environ Int 112:127–133 29272776 10.1016/j.envint.2017.11.020

[8] Chen D, Kannan K, Tan H, Zheng Z, Feng YL, Wu Y et al (2016) Bisphenol analogues other than BPA: environmental occurrence, human exposure, and Toxicity - A review. Environ Sci Technol 50(11):5438–5453 27143250 10.1021/acs.est.5b05387

[9] Liao C, Liu F, Alomirah H, Loi VD, Mohd MA, Moon HB et al (2012) Bisphenol S in urine from the united States and seven Asian countries: occurrence and human exposures. Environ Sci Technol 46(12):6860–6866 22620267 10.1021/es301334j

[10] The use of bisphenol A and its alternatives in thermal paper in the EU during 2014–2022 [Internet] (2020) Available from: http://echa.europa.eu/contact

[11] Lehmler HJ, Liu B, Gadogbe M, Bao W (2018) Exposure to bisphenol A, bisphenol F, bisphenol S in U.S. Adults and children: the National health and nutrition examination survey 2013–2014. ACS Omega 3(6):6523–6532 29978145 10.1021/acsomega.8b00824 PMC6028148

[12] Philips EM, Jaddoe VWV, Asimakopoulos AG, Kannan K, Steegers EAP, Santos S et al (2018) Bisphenol and phthalate concentrations and its determinants among pregnant women in a population-based cohort in the Netherlands, 2004–5. Environ Res 161:562–572 29245124 10.1016/j.envres.2017.11.051 PMC5820024

[13] Dimitriadis I, Minguez-Alarcon L, Williams P, Souter I, Toth TL, Ford JB et al (2017) Follicular fluid (FF) phenol concentrations and early in vitro fertilization (IVF) outcomes among women seeking fertility care. Fertil Steril 108(3):e89

[14] Thayer KA, Taylor KW, Garantziotis S, Schurman SH, Kissling GE, Hunt D et al (2016) Bisphenol A, bisphenol S, and 4-hydro Xyphenyl 4-isopro oxyphenyl sulfone (bpsip) in urine and blood of cashiers. Environ Health Perspect 124(4):437–444 26309242 10.1289/ehp.1409427 PMC4824622

[15] Zimmers SM, Browne EP, O’Keefe PW, Anderton DL, Kramer L, Reckhow DA et al (2014) Determination of free bisphenol A (BPA) concentrations in breast milk of U.S. Women using a sensitive LC/MS/MS method. Chemosphere 104:237–243 24507723 10.1016/j.chemosphere.2013.12.085

[16] Song Y, Xie P, Cai Z (2018) Metabolism of bisphenol S in mice after oral administration. Rapid Commun Mass Spectrom 32(6):495–502 29280213 10.1002/rcm.8051

[17] Wu LH, Zhang XM, Wang F, Gao CJ, Chen D, Palumbo JR et al (2018) Occurrence of bisphenol S in the environment and implications for human exposure: A short review. Sci Total Environ 615:87–98 28963899 10.1016/j.scitotenv.2017.09.194

[18] Eladak S, Grisin T, Moison D, Guerquin MJ, N’Tumba-Byn T, Pozzi-Gaudin S et al (2015) A new chapter in the bisphenol a story: bisphenol S and bisphenol F are not safe alternatives to this compound. Fertil Steril 103(1):11–21 25475787 10.1016/j.fertnstert.2014.11.005

[19] Naderi M, Wong MYL, Gholami F (2014) Developmental exposure of zebrafish (Danio rerio) to bisphenol-S impairs subsequent reproduction potential and hormonal balance in adults. Aquat Toxicol 148:195–203 24508763 10.1016/j.aquatox.2014.01.009

[20] Ahsan N, Ullah H, Ullah W, Jahan S (2018) Comparative effects of bisphenol S and bisphenol A on the development of female reproductive system in rats; a neonatal exposure study. Chemosphere 197:336–343 29407803 10.1016/j.chemosphere.2017.12.118

[21] Hill CE, Sapouckey SA, Suvorov A, Vandenberg LN (2017) Developmental exposures to bisphenol S, a BPA replacement, alter estrogen-responsiveness of the female reproductive tract: a pilot study HHS public access. Cogent Med 4(1):1317690 31231671 PMC6588183

[22] Ji K, Hong S, Kho Y, Choi K (2013) Effects of bisphenol S exposure on endocrine functions and reproduction of zebrafish. Environ Sci Technol 47(15):8793–8800 23806087 10.1021/es400329t

[23] Pu Y, Gingrich JD, Steibel JP, Veiga-Lopez A (2017) Sex-specific modulation of fetal adipogenesis by gestational bisphenol A and bisphenol S exposure. Endocrinology 158(11):3844–3858 28938450 10.1210/en.2017-00615 PMC5695840

[24] Qiu W, Zhao Y, Yang M, Farajzadeh M, Pan C, Wayne NL (2016) Actions of bisphenol A and bisphenol S on the reproductive neuroendocrine system during early development in zebrafish. Endocrinology 157(2):636–647 26653335 10.1210/en.2015-1785

[25] Catanese MC, Vandenberg LN (2017) Bisphenol S (BPS) alters maternal behavior and brain in mice exposed during pregnancy/lactation and their daughters. Endocrinology 158(3):516–530 28005399 10.1210/en.2016-1723 PMC5460783

[26] Crump D, Chiu S, Williams KL (2016) Bisphenol S alters embryonic viability, development, gallbladder size, and messenger RNA expression in chicken embryos exposed via egg injection. Environ Toxicol Chem 35(6):1541–1549 26606162 10.1002/etc.3313

[27] Ullah H, Jahan S, Ain QU, Shaheen G, Ahsan N (2016) Effect of bisphenol S exposure on male reproductive system of rats: A histological and biochemical study. Chemosphere 152:383–391 26994432 10.1016/j.chemosphere.2016.02.125

[28] Lin M, Hua R, Ma J, Zhou Y, Li P, Xu X et al (2021) Bisphenol A promotes autophagy in ovarian granulosa cells by inducing AMPK/mTOR/ULK1 signaling pathway. Environ Int 147:106298 33387880 10.1016/j.envint.2020.106298

[29] Aftabsavad S, Noormohammadi Z, Moini A, Karimipoor M (2021) Effect of bisphenol A on alterations of ICAM-1 and HLA-G genes expression and DNA methylation profiles in cumulus cells of infertile women with poor response to ovarian stimulation. Sci Rep 11(1):9595 33953208 10.1038/s41598-021-87175-1 PMC8099902

[30] Yenigül NN, Dilbaz S, Dilbaz B, Kaplanoğlu İ, Güçel F, Aldemir O et al (2021) The effect of plastic bottled water consumption on outcomes of ICSI cycles undertaken for unexplained infertility. Reprod Biomed Online 43(1):91–99 34001442 10.1016/j.rbmo.2021.04.010

[31] Ferris J, Mahboubi K, MacLusky N, King WA, Favetta LA (2016) BPA exposure during in vitro oocyte maturation results in dose-dependent alterations to embryo development rates, apoptosis rate, sex ratio and gene expression. Reprod Toxicol 59:128–138 26686065 10.1016/j.reprotox.2015.12.002

[32] Ferris J, Favetta LA, King WA (2015) Bisphenol A exposure during oocyte maturation in vitro results in spindle abnormalities and chromosome misalignment in Bos taurus. Cytogenet Genome Res 145(1):50–58 25871885 10.1159/000381321

[33] Karwacka A, Zamkowska D, Radwan M, Jurewicz J (2019) Exposure to modern, widespread environmental endocrine disrupting chemicals and their effect on the reproductive potential of women: an overview of current epidemiological evidence. Hum Fertil 22(1):2–25 10.1080/14647273.2017.1358828 28758506

[34] Machtinger R, Orvieto R (2014) Bisphenol A, oocyte maturation, implantation, and IVF outcome: review of animal and human data. Reprod Biomed Online 29(4):404–410 25154017 10.1016/j.rbmo.2014.06.013

[35] Liu J, Li J, Wu Y, Zhao Y, Luo F, Li S et al (2017) Bisphenol A metabolites and bisphenol S in paired maternal and cord serum. Environ Sci Technol 51(4):2456–2463 28110528 10.1021/acs.est.6b05718

[36] Zhang B, He Y, Zhu H, Huang X, Bai X, Kannan K et al (2020) Concentrations of bisphenol A and its alternatives in paired maternal–fetal urine, serum and amniotic fluid from an e-waste dismantling area in China. Environ Int 136:105407 31955035 10.1016/j.envint.2019.105407

[37] Directive (2010) /63/EU of the European Parliament and of the Council on the protection of animals used for scientific purposes. Official Journal of the European Union L 276, 20 October 2010, pp. 33–79

[38] Russell WM StrattoN, Rex Leonard Burch (1959) The principles of humane experimental technique. Methuen

[39] Percie du Sert N, Hurst V, Ahluwalia A, Alam S, Avey MT, Baker M et al (2020) The ARRIVE guidelines 2.0: updated guidelines for reporting animal research. J Cereb Blood Flow Metab 40(9):1769–1777 32663096 10.1177/0271678X20943823 PMC7430098

[40] Smith AJ (2020) Guidelines for planning and conducting high-quality research and testing on animals. Lab Anim Res 10:3621 10.1186/s42826-020-00054-0 PMC7348107 32665911

[41] Peretz J, Craig ZR, Flaws JA (2012) Bisphenol A inhibits follicle growth and induces Atresia in cultured mouse antral follicles independently of the genomic estrogenic pathway. Biol Reprod 87(3):63 22743301 10.1095/biolreprod.112.101899 PMC3464906

[42] Saleh AC, Sabry R, Mastromonaco GF, Favetta LA (2021) BPA and BPS affect the expression of anti-Mullerian hormone (AMH) and its receptor during bovine oocyte maturation and early embryo development. Reproductive Biology Endocrinol 19(1):119 10.1186/s12958-021-00773-6 PMC8330045 34344364

[43] Nevoral J, Kolinko Y, Moravec J, Žalmanová T, Hošková K, Prokešová Š et al (2018) Long-term exposure to very low doses of bisphenol S affects female reproduction. Reproduction 156(1):47–57 29748175 10.1530/REP-18-0092

[44] Nourian A, Soleimanzadeh A, Jalali AS, Najafi G (2017) Effects of bisphenol-S low concentrations on oxidative stress status and in vitro fertilization potential in mature female mice. Vet Res Forum 8(4):341–345 29326794 PMC5756255

[45] Desmarchais A, Téteau O, Kasal-Hoc N, Cognié J, Lasserre O, Papillier P et al (2022) Chronic low BPS exposure through diet impairs in vitro embryo production parameters according to metabolic status in the Ewe. Ecotoxicol Environ Saf 229:113096 34952380 10.1016/j.ecoenv.2021.113096

[46] Harnett KG, Moore LG, Chin A, Cohen IC, Lautrup RR, Schuh SM (2021) Teratogenicity and toxicity of the new BPA alternative TMBPF, and BPA, BPS, and BPAF in chick embryonic development. Curr Res Toxicol 2:399–410 34901887 10.1016/j.crtox.2021.11.001 PMC8639335

[47] Panagopoulos P, Mavrogianni D, Christodoulaki C, Drakaki E, Chrelias G, Panagiotopoulos D et al (2023) Effects of endocrine disrupting compounds on female fertility. Best Pract Res Clin Obstet Gynaecol 88:102347 37244786 10.1016/j.bpobgyn.2023.102347

[48] Choi BI, Harvey AJ, Green MP (2016) Bisphenol A affects early bovine embryo development and metabolism that is negated by an oestrogen receptor inhibitor. Sci Rep 6:29318 27384909 10.1038/srep29318 PMC4935887

[49] Arancio AL, Cole KD, Dominguez AR, Cohenour ER, Kadie J, Maloney WC et al (2019) Bisphenol A, bisphenol AF, di-n-butyl phthalate, and 17β-estradiol have shared and unique dose-dependent effects on early embryo cleavage divisions and development in Xenopus laevis. Reprod Toxicol 84:65–74 30579998 10.1016/j.reprotox.2018.12.005

[50] Cohen IC, Cohenour ER, Harnett KG, Schuh SM (2021) BPA, BPAF and TMBPF alter adipogenesis and fat accumulation in human mesenchymal stem cells, with implications for obesity. Int J Mol Sci 22(10):5363 34069744 10.3390/ijms22105363 PMC8160667

[51] Kojima H, Takeuchi S, Sanoh S, Okuda K, Kitamura S, Uramaru N et al (2019) Profiling of bisphenol A and eight its analogues on transcriptional activity via human nuclear receptors. Toxicology 413:48–55 30582956 10.1016/j.tox.2018.12.001

[52] Liang S, Yin L, Yu KS, Hofmann MC, Yu X (2017) High-content analysis provides mechanistic insights into the testicular toxicity of bisphenol A and selected analogues in mouse spermatogonial cells. Toxicol Sci 155(1):43–60 27633978 10.1093/toxsci/kfw178 PMC5216646

[53] Michałowicz J, Mokra K, Bak A (2015) Bisphenol A and its analogs induce morphological and biochemical alterations in human peripheral blood mononuclear cells (in vitro study). Toxicol in Vitro 29(7):1464–1472 26028149 10.1016/j.tiv.2015.05.012

[54] Moreman J, Lee O, Trznadel M, David A, Kudoh T, Tyler CR (2017) Acute toxicity, teratogenic, and estrogenic effects of bisphenol A and its alternative replacements bisphenol S, bisphenol F, and bisphenol AF in zebrafish Embryo-Larvae. Environ Sci Technol 51(21):12796–12805 29016128 10.1021/acs.est.7b03283

[55] Mina A, Boutzios G, Papoutsis I, Kaparos G, Christopoulos P, Kousta E et al (2022) Bisphenol A correlates with fewer retrieved oocytes in women with tubal factor infertility. Hormones 21(2):305–315 35524040 10.1007/s42000-022-00370-1

[56] Ehrlich S, Williams PL, Missmer SA, Flaws JA, Ye X, Calafat AM et al (2012) Urinary bisphenol A concentrations and early reproductive health outcomes among women undergoing IVF. Hum Reprod 27(12):3583–3592 23014629 10.1093/humrep/des328 PMC3501244

[57] Vrachnis N, Loukas N, Vrachnis D, Antonakopoulos N, Zygouris D, Kolialexi A et al (2021) A systematic review of bisphenol a from dietary and non-dietary sources during pregnancy and its possible connection with fetal growth restriction: investigating its potential effects and the window of fetal vulnerability. Nutrients 13(7):2426 34371934 10.3390/nu13072426 PMC8308698

[58] Goldinger DM, Demierre AL, Zoller O, Rupp H, Reinhard H, Magnin R et al (2015) Endocrine activity of alternatives to BPA found in thermal paper in Switzerland. Regul Toxicol Pharmacol 71(3):453–462 25579646 10.1016/j.yrtph.2015.01.002

[59] Rochester JR, Bolden AL (2015) Bisphenol S and F: A systematic review and comparison of the hormonal activity of bisphenol a substitutes. Environ Health Perspect 123(7):643–650 25775505 10.1289/ehp.1408989 PMC4492270

[60] Roelofs MJE, Berg M, van den, Bovee TFH, Piersma AH, van Duursen MBM (2015) Structural bisphenol analogues differentially target steroidogenesis in murine MA-10 Leydig cells as well as the glucocorticoid receptor. Toxicology 329:10–20 25576683 10.1016/j.tox.2015.01.003

[61] Rosenmai AK, Dybdahl M, Pedersen M, van Vugt-Lussenburg BMA, Wedebye EB, Taxvig C et al (2014) Are structural analogues to bisphenol a safe alternatives? Toxicol Sci 139(1):35–47 24563381 10.1093/toxsci/kfu030

[62] Siracusa JS, Yin L, Measel E, Liang S, Yu X (2018) Effects of bisphenol A and its analogs on reproductive health: A mini review. Reprod Toxicol 79:96–123 29925041 10.1016/j.reprotox.2018.06.005 PMC6689411

[63] Jin H, Zhu J, Chen Z, Hong Y, Cai Z (2018) Occurrence and partitioning of bisphenol analogues in adults’ blood from China. Environ Sci Technol 16(2):812–820 10.1021/acs.est.7b03958 29243481

[64] Wan Y, Xia W, Yang S, Pan X, He Z, Kannan K (2018) Spatial distribution of bisphenol S in surface water and human serum from Yangtze river watershed, China: implications for exposure through drinking water. Chemosphere 199:595–602 29459349 10.1016/j.chemosphere.2018.02.040

[65] Yamazaki E, Yamashita N, Taniyasu S, Lam J, Lam PK, Moon HB, Jeong Y, Kannan P, Achyuthan H, Munuswamy N, Kannan K (2015) Bisphenol A and other bisphenol analogues including BPS and BPF in surface water samples from Japan, China, Korea and India. Ecotoxicol Environ Saf 122:565–572 26436777 10.1016/j.ecoenv.2015.09.029

[66] Song S, Song M, Zeng L, Wang T, Liu R, Ruan T, Jiang G (2014) Occurrence and profiles of bisphenol analogues in municipal sewage sludge in China. Environ Pollut 186:14–19 24355443 10.1016/j.envpol.2013.11.023

[67] Liao C, Kannan K (2013) Concentrations and profiles of bisphenol A and other bisphenol analogues in foodstuffs from the united States and their implications for human exposure. J Agric Food Chem 61:4655–4662 23614805 10.1021/jf400445n

[68] Vinas P, Campillo N, Martinez-Castillo N, Hernandez-Cordoba M (2010) Comparison of two derivatization-based methods for solid-phase microextraction-gas chromatography-mass spectrometric determination of bisphenol A, bisphenol S and biphenol migrated from food cans. Anal Bioanal Chem 397:115–125 20127078 10.1007/s00216-010-3464-7

[69] Liao C, Liu F, Guo Y, Moon HB, Nakata H, Wu Q, Kannan K (2012) Occurrence of eight bisphenol analogues in indoor dust from the united States and several Asian countries: implications for human exposure. Environ Sci Technol 46:9138–9145 22784190 10.1021/es302004w

[70] Skledar DG, Schmidt J, Fic A, Klopčič I, Trontelj J, Dolenc MS, Finel M, Mašič LP (2016) Influence of metabolism on endocrine activities of bisphenol S. Chemosphere 157(Supplement C) 152–159 10.1016/j.chemosphere.2016.05.027 27213244

[71] Dreier DA, Connors KA, Brooks BW (2015) Comparative endpoint sensitivity of in vitro Estrogen agonist assays. Regul Toxicol Pharmacol 72(2):185–193 25896097 10.1016/j.yrtph.2015.04.009

[72] Huang R, Sakamuru S, Martin MT, Reif DM, Judson RS, Houck KA, Casey W, Hsieh JH, Shockley KR, Ceger P, Fostel J, Witt KL, Tong W, Rotroff DM, Zhao T, Shinn P, Simeonov A, Dix DJ, Austin CP, Kavlock RJ, Tice RR, Xia M (2014) Profiling of the Tox21 10K compound library for agonists and antagonists of the Estrogen receptor alpha signaling pathway. Sci Rep 4:5664 25012808 10.1038/srep05664 PMC4092345

[73] Rochester JR, Bolden AL (2015) Bisphenol S and F: A systematic review and comparison of the hormonal activity of bisphenol A substitutes. Environ Health Persp 123(7):643–650 10.1289/ehp.1408989 PMC4492270 25775505

[74] Chang KT, Su YT, Tsai YR, Lan KC, Hsuuw YD, Kang HY, Chan WH, Huang FJ (2022) High levels estradiol affect blastocyst implantation and post-implantation development directly in mice. Biomed J 45(1):179–189 35148258 10.1016/j.bj.2021.01.004 PMC9133257

[75] Téteau O, Jaubert M, Desmarchais A, Papillier P, Binet A, Maillard V et al (2020) Bisphenol A and S impaired ovine granulosa cell steroidogenesis. Reproduction 159(5):571–583 από 57 32092037 10.1530/REP-19-0575

[76] Assou S, Boumela I, Haouzi D, Anahory T, Dechaud H, DeVos J, Hamamah S (2011) Dynamic changes in gene expression during human early embryo development: from fundamental aspects to clinical applications. Hum Reprod Update 17(2):272–290 20716614 10.1093/humupd/dmq036 PMC3189516

[77] Zhang MY, Tian Y, Yan ZH, Li WD, Zang CJ, Li L, Sun XF, Shen W, Cheng SF (2020) Maternal bisphenol S exposure affects the reproductive capacity of F1 and F2 offspring in mice. Environ Pollut 267:115382 32866863 10.1016/j.envpol.2020.115382

